# Leptin: a gender and obesity-related marker predictive of metabolic comorbidities and therapeutic response to anti-IL-23 biologic drugs in psoriatic patients

**DOI:** 10.3389/fimmu.2025.1607312

**Published:** 2025-07-16

**Authors:** Roberta Belli, Anna Dattolo, Francesca Sampogna, Emanuela Gubinelli, Daniela Lulli, Gaia Moretta, Emanuele Scala, Luca Sanna, Matteo Megna, Maria Vittoria Cannizzaro, Melania Parisi, Cecilia Luordi, Claudia Scarponi, Maria Quaranta, Maria Grazia Lolli, Lorena Silvestri, Paolo Gisondi, Giampiero Girolomoni, Sabatino Pallotta, Cristina Albanesi, Laura Mercurio, Stefania Madonna

**Affiliations:** ^1^ Laboratory of Experimental Immunology, Istituto Dermopatico dell'Immacolata IDI-IRCCS, Rome, Italy; ^2^ Istituto Dermopatico dell'Immacolata IDI-IRCCS, Rome, Italy; ^3^ Department of Dermatology, Istituto Dermopatico dell'Immacolata IDI-IRCCS, Rome, Italy; ^4^ Section of Dermatology - Department of Clinical Medicine and Surgery, University of Naples Federico II, Naples, Italy; ^5^ Dermatology Section, Department of Medicine, University of Verona, Verona, Italy

**Keywords:** psoriasis, obesity, adipokines, anti-IL-17 biologics, anti-IL-23 biologics, gender

## Abstract

**Introduction:**

Psoriasis is a chronic immune-mediated inflammatory skin disorder, frequently associated with comorbidities such as obesity, which can exacerbate its severity and hinder treatment efficacy. Psoriasis pathogenesis involves complex interactions among genetic, environmental, hormonal factors, and is characterized by dysregulated immune responses. In this study, we investigated the relationship between obesity and psoriasis, exploring the impact of circulating levels of adipokines on disease severity, comorbidities, and treatment response to anti-IL-17 and anti-IL-23 biologics.

**Methods:**

We conducted an observational study that included 91 patients with psoriasis eligible for biological therapy, as well as 26 healthy controls. Disease severity was assessed using PASI, along with the measurement of body composition. Serum samples were analyzed for the measurement of adipokine levels and lipid profiles. Clinical parameters, bioelectrical impedance analysis (BIA), serum adipokine levels (leptin, visfatin, adiponectin) and lipid profile were assessed at baseline and after 16 weeks of biologic treatments.

**Results:**

Clinical parameters and adiposity-related indices were analyzed in 76 patients at both T0 and 16 weeks of anti-IL-17 and anti-IL-23 biological treatments, while serum adipokine levels were assessed in 66 patients. Psoriatic patients exhibited higher body mass index (BMI), waist circumference, fat mass (FM), and levels of visfatin (a pro-inflammatory adipokine), whereas adiponectin levels (an anti-inflammatory adipokine) were lower compared to controls. Circulating leptin (a pro-inflammatory adipokine) was significantly higher in female psoriatic patients and showed a positive correlation with the PASI score. Leptin also positively correlated with adiposity indices, while adiponectin showed negative correlations. Furthermore, in women, leptin levels were also associated with psoriatic arthritis, hypertension and, at lower extent, with type II diabetes. Finally, treatment with anti-IL-23 led to a reduction in visfatin levels in female psoriatic patients and resulted in a significant decrease in fat mass percentage in men. Notably, higher baseline leptin levels were associated with the failure to achieve an 90% improvement in baseline PASI at W16 of anti-IL-23 biologic treatments.

**Conclusions:**

This study highlights significant sex-specific differences in the relationships between adipokines, body composition indices, psoriasis severity, comorbidities, and clinical outcome to therapies. Leptin, in particular, may serve as a predictive biomarker for response to anti-IL-23 therapies.

## 1 Introduction

Psoriasis is an immune-mediated, inflammatory disease, with a global prevalence of 2–3%. It occurs equally in men and women but poses an exceptionally high burden for women (1).

The pathogenesis of psoriasis involves genetic, environmental, hormonal, and immune factors. There is an altered interplay between the innate and adaptive arms of the immune system, which leads to the activation of T helper (Th) 1, Th17 and Th22 lymphocytes, resulting in the production of large amounts of pro-inflammatory cytokines, including interferon (IFN)-γ, tumor necrosis factor (TNF)-α, interleukin (IL)-17, and IL-22 (2). Despite strong hereditary factors, exogenous stimuli such as infection, trauma, and stress play a significant role in disease manifestation (3). Hormonal factors also affect psoriasis, with high estrogen levels correlating with its improvement (1). Psoriasis can be associated with a number of comorbidities such as obesity, diabetes, metabolic syndrome (MetS), inflammatory bowel diseases, psoriatic arthritis (PsA), cardiovascular diseases, and psychiatric disorders. Several studies have identified obesity as a risk factor for the development of psoriasis in both women and men and have associated it with a more severe psoriasis phenotype (4–7).

Obesity is defined as an increased fat deposition determined by the combination of genetic factors, decreased physical activity, and over-consumption of energy-dense food (8) and is characterized by chronic low-grade inflammation (9). The exact mechanism responsible for the association between psoriasis and obesity is not yet completely understood (10). Indeed, a few association studies have relied only on BMI to determine obesity, even though such a measure fails to distinguish body components such as fat mass and free fat mass in individuals (8). There is evidence that patients with psoriasis have an increased visceral adiposity, which represents not only an energy deposit, but also an endocrine organ secreting adipokines, including leptin, visfatin, adiponectin, resistin, chemerin, as well as pro-inflammatory mediators such as IL-6 and TNF-α, all with different immunomodulatory properties (5, 11). In particular, leptin has a pro-inflammatory role in immune responses, as it modulates monocyte differentiation into M1 macrophages and induces Th1 polarization of lymphocytes (12).

Leptin expression is increased in individuals with central obesity and type-2 diabetes (13, 14) and is mainly driven by the IL-23/Th17 axis in psoriatic patients (15). Similarly, visfatin activates human leukocytes and induces cytokine production. In CD14^+^ monocytes, visfatin stimulates the production of IL-1, TNF-α, and IL-6, while also increasing the surface expression of costimulatory molecules CD54, CD40, and CD80 (16).

Unlike leptin, visfatin expression is strongly induced by Th1/Th17-cytokines and also acts on keratinocytes by inducing hyperproliferation and impairing their terminal differentiation (17). In contrast to leptin and visfatin, adiponectin is known to have an anti-inflammatory role, being able to modulate monocyte function favouring the M2 phenotype and counteracting the polarization of Th1 and Th17lymphocytes. Indeed, it has been shown to have a protective effect against the development of psoriasis (18, 19).

Dysregulated levels of circulating adipokines have been reported in both psoriatic and in obese patients (20). In fact, studies have shown that patients with psoriasis tend to have higher serum levels of TNF-α, IL-6, and resistin (21). Conversely, levels of anti-inflammatory adipokines such as adiponectin may be lower in these patients (22). This imbalance can further promote inflammation in psoriasis. Similar to other comorbidities, the presence of obesity interferes with the effectiveness of the treatments for psoriasis. Indeed, obesity is a negative predictor of efficacy for several systemic drugs, in particular those with a fixed dosage, particulalry biologics. In addition to TNF-α inhibitors, biologics approved for moderate-to-severe psoriasis include IL-12/23 inhibitors (Ustekinumab), IL-17 inhibitors (Ixekizumab, Secukinumab, Brodalumab, Bimekizumab), and IL-23 inhibitors (Guselkumab, Risankizumab, Tildrakizumab) (23). Obesity negatively affects the clinical response of biological drugs in psoriatic patients, with differences among different biologic classes and drugs (4, 23, 24). Adipokines released by adipose tissue may interfere with the mechanisms of action of biologic therapies (25).

In this study, we investigated the circulating levels of adipokines, particularly of leptin, visfatin and adiponectin in psoriatic patients, and their association with disease severity and presence of high-prevalence comorbidities. We further explored the associations between adipokine levels and body composition parameters and lipid profile in our cohort of psoriasis patients. Finally, we investigated the impact of anti-IL-17 and anti-IL-23 biologics on adipokine levels, body composition indices, and lipid profile.

## 2 Materials and methods

### 2.1 Study population

This was a multicenter, observational, longitudinal study that included adult patients with moderate-to-severe chronic plaque psoriasis. A case-control study was also performed. Patients were recruited between September 2023 and October 2024 at the Dermatology Units of IDI-IRCCS in Rome, Azienda Ospedaliera of Naples Federico II, and Dermatology and Venereology, Department of Medicine, Azienda Ospedaliera of Verona, Italy. This study adhered to the ethical guidelines of the World Medical Association (Declaration of Helsinki) for the collection of blood-derived serum samples from patients and was approved by the Ethical Committee of IDI-IRCCS (721/CE/2024). All participants provided written informed consent after receiving a clear explanation of the aim of the study.

Inclusion criteria were as follows: patients with a diagnosis of chronic plaque psoriasis; age > 18 years; Psoriasis Area Severity Index (PASI) score ≥ 10; and body surface area (BSA) ≥ 10%. Patients with a baseline PASI < 10, who presented involvement of sensitive areas (scalp, hands, face, nails or genitals), were also included. All patients involved in the study started treatment with an anti-IL-17A (Secukinumab, Ixekizumab), anti-IL17A and F (Bimekizumab) or anti-IL-23 (Guselkumab, Risankizumab, and Tildrakizumab) biologic drugs, according to physician discretion and standard clinical practice. Biologic drugs were administered according to Agenzia Italiana del Farmaco (AIFA) criteria, using standard dosing regimens (anti-IL-17 drugs: Secukinumab - 300 mg dosage, Ixekizumab – 160 mg dosage at week 0 than 80 mg, Bimekizumab – 320 mg dosage; anti IL-23 drugs: Guselkumab – 100 mg dosage; Risankizumab 150 mg dosage; Tildrakizumab - 100-mg dosage for patients with body weight < 90 kg, 200-mg dosage for those with a weight > 90 kg) in monotherapy without concurrent conventional systemic or topical therapies (4).

Exclusion criteria included: patients undergoing systemic or biologic therapies for psoriasis in the three weeks prior to the date of enrollment, and patients diagnosed with severe psychiatric disorders.

Control subjects were chosen among hospital volunteers and were matched to psoriatic patients by age, sex, and BMI.

### 2.2 Anthropometric and clinical data collection

At the first visit, demographic data including age, sex, and ethnicity were collected for each patient. Clinical parameters were also recorded, including age of psoriasis onset, family history of psoriasis, disease severity, assessed by PASI score, body surface area (BSA), and presence of nail involvement.

For each patient, personal data, as well as anthropometric, sociodemographic and clinical data were collected and annotated in a database *ad hoc* created for the study. The disease severity and response to treatment were evaluated using the PASI score at baseline (T0) and at week 16 (W16) of treatment.

Clinical efficacy was defined as the 75%, 90%, and 100% improvement of PASI score compared with baseline, referred to as PASI75, PASI90 and PASI100, respectively.

In addition, during the first visit the presence of comorbidities such as Psoriatic Arthritis (PsA), type 2 diabetes, hypertension, and cardiovascular disease (including myocardial infarction, acute coronary syndrome, mitral valve prolapse, and bradycardia) was documented.

Finally, we recorded weight, height, waist circumference (WC), and BMI, calculated as the ratio between weight and square of height (kg/m^2^). Subjects were classified into three classes: normal weight (BMI between 18.5 and 24.9kg/m^2^), overweight (BMI between 25 and 29.9kg/m^2^), and obese (BMI ≥ 30kg/m^2^), according to the World Health Organization (WHO) criteria .

Blood samples were collected from all study participants in EDTA tubes under fasting conditions for serum fraction isolation at T0 and W16 of treatment for the analysis of adipokines and lipid profile parameters (see 2.4 paragraph).

### 2.3 Bioelectrical impedance analysis

During the body composition examination, participants stood on the Accuniq BC380 impedance scale (Selvas Healthcare Inc. Republic of Korea) with their arms slightly open and touched eight electrodes with their hands and feet according to the manufacturer’s instructions. Subjects wore light clothing, removed shoes and socks, and had the electrode contact areas scrubbed with alcohol before each application. Participants fasted for at least 2 hours prior to the test. All patients performed the BIA analysis at T0 and W16 of treatment. The same operator conducted all measurements. Through impedance measurement, BIA enables the assessment of whole-body and segmental (arms, legs, trunk) composition, in terms of water, protein, minerals, fat mass, and lean mass. The BIA parameters considered for association analysis in this study were: percentage of fat mass, visceral fat assessed as Visceral Fat Level (VFL) and Visceral Fat Area (VFA), and waist circumference. Fat mass refers to the total amount of body fat within an individual, consisting of subcutaneous and visceral fat. VFA and VFL are key parameters for evaluating obesity and its associated metabolic complications . VFA quantitatively measures the volume of visceral fat, while VFL is a relative measure of visceral fat compared to a reference range.

### 2.4 Serum samples collection and determination of adipokine concentrations by enzyme linked immunosorbent assay

Blood samples were collected from patients and control subjects in the morning after an overnight fast, into K2EDTA vacutainers and centrifuged at 1700 RCF for 20 minutes. Resulting serum was then separated and stored at -80°C for subsequent adipokine analysis. On the same day, an aliquot of blood was processed to determine lipid profile parameters, including total cholesterol, low-density lipoprotein (LDL), high-density lipoprotein (HDL), and triglycerides (TG). The Visceral Adiposity Index (VAI) score was calculated using the following sex-specific formula, where WC=waist circumference, BMI=Body Mass Index, TG=triglycerides (expressed in mmol/L), and HDL=high-density lipoprotein (expressed in mmol/L):

Males: [WC/39.68 + (1.88 x BMI)] x (TG/1.03) x (1.31/HDL);

Females: [WC/36.58 + (1.89 x BMI)] x [TG/0.81] x (1.52/HDL) (26).

Serum levels of the adipokines visfatin, leptin and adiponectin were quantified by the enzyme-linked immunosorbent assay kit (ELISA kit: Visfatin AG-45A-000-6Y TP-KIO1, Adipogen; Leptin RD 191001100, BioVendor; Human Total Adiponectin/Acrp30 Quantikine R&D, respectively), according to user’s manual. The plates were analyzed using an ELISA reader (model 3550 UV; Bio-Rad, Hercules, CA, USA). The detection limits of the assays (detection threshold) were 30 ng/mL for visfatin, 0.2 ng/mL for leptin and 0.891 ng/mL for total adiponectin.

### 2.5 Statistical analysis

Categorical variables were described as number (n) and percentage (%), and continuous variables as mean ± standard deviation. Categorical variables were compared in different groups using Fisher’s exact test, and continuous variables using Mann-Whitney *U* test for two groups and the Kruskal-Wallis test for more than two groups. Mean values at baseline and at week 16 were compared using Wilcoxon non-parametric test. Box plots were used to represent the distribution of some variables, highlighting the median value and the interquartile range. Correlations among variables was calculated using Spearman’s rho correlation coefficient. A linear regression model was tested to identify the variables associated with PASI. Independent variables were those positively correlated to PASI in the univariate analysis and the three adipokines. Multicollinearity among variables was verified measuring the Variance Inflation Factor (VIF). A logistic regression model was tested with PASI90 at week 16 as the dependent variable, and sex, BMI, baseline leptin levels, and treatment type as independent variables. In all statistical analyses a *p*-value <0.05 was considered significant. All analyses were performed using SPSS Statistical package. Graphs were performed with GraphPad-Prism Version 8.0.

## 3 Results

### 3.1 Characteristics of study population

The study population consisted of 117 participants, 91 cases (63.7% males), and 26 controls (42.3% males). The mean ± standard deviation age of patients was 47.5 ± 17 years, whereas mean age of controls was 44.1 ± 11.5 years. The most common comorbidities among patients were Psoriatic Arthritis (PsA, 27.5%), diabetes (11%), and hypertension (27.5%). **Table 1A** reports the anthropometric data, BIA indices, and presence of comorbidities in the total population, as well as the same data stratified by sex. Anthropometric data and BIA indices were analysed in 76 (83.5%) of 91 patients at both T0 and W16, while serum adipokine levels were assessed in 66 (72.5%) patients. A flowchart presented in **Supplementary Figure 1** illustrates the number of patients analyzed during the various steps and the rationale for their sequential exclusion. Men had a significantly higher mean waist circumference (cm) than women (**Table 1A**). Percentage of VFA tended to be also higher in men than in women (145.2 ± 99.8 vs 104.0 ± 73.9), although the difference was not significant (*p*= 0.054). Women had mean levels of both HDL cholesterol and leptin significantly higher than those observed in men (**Table 1A**). Mean of BMI values (kg/m^2^) were comparable between men and women (27.7 ± 5.5 vs 27.5 ± 6.5). Similar differences between males and females were also present in the control group (**Table 1B**). Men showed mean values (cm) for waist circumference, VFA, and VFL, significantly higher than women (**Table 1A**). Similar to the case group, control female subjects showed higher leptin levels than men (**Table 1B**). Finally, BMI was comparable between male and female control subjects (25.4 ± 4.5 kg/m^2^ vs 24.1 ± 4.1kg/m^2^).

**Table 1A:** Participant’s characteristics.

	TOTAL (n=91)	MEN (n=58)	WOMEN (n=33)	
Variable	Mean (SD)	Mean (SD)	Mean (SD)	p*
Age (years)	47.5 (17.1)	48.4 (17.8)	45.9 (15.8)	*0.504*
Age at onset (years)	27.9 (17.8)	30.1 (17.6)	23.8 (17.7)	*0.061*
Body Mass Index (BMI, kg/m^2^)	27.6 (5.9)	27.7 (5.5)	27.5 (6.5)	*0.647*
Baseline PASI	15.4 (10.4)	16.1 (11.7)	14.1 (7.8)	*0.710*
Waist circumference (cm)	95.1 (16.8)	98.5 (16.6)	89.3 (15.8)	***0.010***
Fat mass (%)	33.8 (21.2)	34.1 (23.2)	33.3 (18.0)	*0.618*
Total cholesterol (mg/dL)	181.2 (36.7)	180.1 (38.4)	183.0 (34.0)	*0.697*
HDL cholesterol (mg/dL)	55.5 (16.4)	52.9 (17.2)	59.8 (14.0)	***0.008***
LDL cholesterol (mg/dL)	105.1 (32.1)	107.4 (34.8)	101.1 (26.7)	*0.282*
Triglycerides (mg/dL)	110.0 (55.5)	112.1 (54.0)	106.2 (58.7)	*0.351*
Visceral Fat Area (VFA, cm^2^)	129.4 (92.4)	145.2 (99.8)	104.0 (73.9)	*0.058*
Visceral Fat Level (VFL)	9.9 (4.0)	9.9 (3.9)	9.8 (4.3)	*0.982*
Visfatin (ng/ml)	5.6 (3.7)	5.5 (4.2)	5.7 (2.7)	*0.553*
Leptin (ng/ml)	41.5 (43.7)	28.9 (29.6)	64.8 (52.4)	***<0.001***
Adiponectin (µg/ml)	7.7 (5.0)	6.7 (3.6)	9.3 (6.4)	*0.198*
	N (%)	N (%)	N (%)	p**
**Sex** men women	58 (63.7) 33 (36.3)			
**Comorbidities** arthropathy type 2 diabetes hypercholesterolemia hypertension renal failure cardiovascular diseases	25 (27.5) 10 (11.0) 4 (4.4) 25 (27.5) 7 (7.7) 4 (4.4)	15 (25.9) 5 (8.6) 3 (5.2) 16 (27.6) 6 (10.3) 3 (5.2)	10 (30.3) 5 (15.2) 1 (3.0) 9 (27.3) 1 (3.0) 1 (3.0)	*0.412* *0.267* *0.540* *0.588* *0.202* *0.540*

Clinical and demographic data, anthropometric measurements, BIA (Bioelectrical impedance analysis) parameters, lipid profile and serum adipokine levels were registered for psoriatic patients (n=91). Data are shown as mean ± SD or number (%). A *p-value* in bold type denotes a significant difference (p < 0.05) p* and p** from Mann-Whitney U test.

**Table 1B:** Participant’s characteristics.

	TOTAL (n=26)	MEN (n=11)	WOMEN (n=15)	
Variable	Mean (SD)	Mean (SD)	Mean (SD)	p*
Age (years)	44.1 (11.5)	48.7 (13.3)	40.7 (9.1)	*0.180*
Body Mass Index (BMI, kg/m^2^)	24.7 (4.2)	25.4 (4.5)	24.15 (4.1)	*0.452*
Waist circumference (cm)	85.9 (13.7)	92.7 (13.6)	77.7 (8.7)	***0.010***
Fat mass (%)	19.2 (7.7)	19.9(9.9)	18.19(7.5)	*0.663*
Visceral Fat Area (VFA, cm^2^)	92.4 (67.9)	124.6 (71.3)	53.11 (38.1)	***0.015***
Visceral Fat Level (VFL)	8.0 (3.7)	9.8 (3.3)	5.89 (3.0)	***0.014***
Visfatin (ng/ml)	3.4 (2.9)	2.9 (2.4)	3.73 (3.1)	*0.472*
Leptin (ng/ml)	29.3 (23.1)	17.3 (14.5)	38.04 (24.8)	***0.021***
Adiponectin (µg/ml)	10. 6 (5.3)	8.5 (3.4)	12.13 (5.9)	*0.079*
	N (%)			
**Sex** men women	11 (42.3) 15 (57.7)			

Demographic data, anthropometric measurements, BIA (Bioelectrical impedance analysis) parameters and serum adipokine levels were registered for healthy subjects (n=26). Data are shown as mean ± SD or number (%). A *p-value* in bold type denotes a significant difference (p < 0.05) p* from Mann-Whitney U test.

### 3.2 Serum leptin and visfatin levels are higher in psoriatic patients, whereas adiponectin levels are lower compared to control group

**Figure 1:**
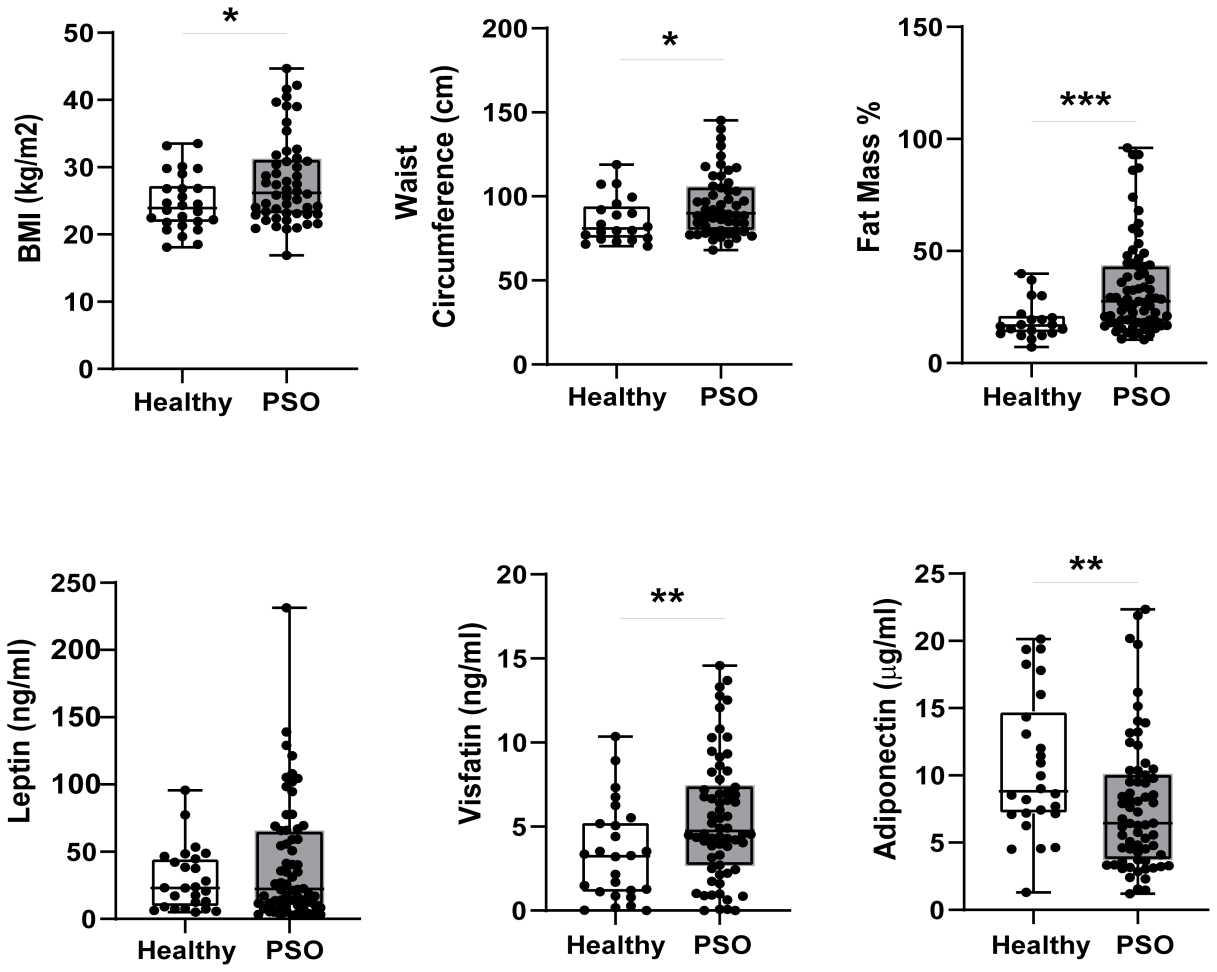
Differences in BIA parameters (n=52 PSO, n=20 Healthy) and circulating adipokine levels (n=66 Pso, n=26 Healthy) between psoriatic patients and control group Results are expressed as boxplots with medians and interquartile ranges ± SD. Difference was analyzed by Mann-Whitney *U* Test. *p-value < 0.05; **p-value < 0.01; ***p-value < 0.001.

First, we explored differences in body composition parameters and serum adipokine levels between psoriatic patients (n = 66) and control subjects (n=26) at baseline. Psoriatic patients exhibited higher values than control subjects for BMI [median 26.20 kg/m^2^ (23.03; 31.28) vs 23.95; *p*= 0.039], waist circumference [89.90 cm (79.35; 105.85) vs 80.85 (74.78; 94.70); *p*= 0.041] and fat mass % [27.60 (18.15; 43.50) vs 16.80 (13.25; 21.50); *p*= 0.001], as shown in **Figure 1**. These findings suggest a higher prevalence of overweight and obesity among psoriatic patients.

Notably, serum visfatin levels were significantly higher in psoriatic patients [4.73 ng/mL (2.66;7.46)] than in control subjects [3.25 ng/mL (1.06;5.27); *p*= 0.010], whereas leptin content tended to be higher in psoriatic patients compared to the healthy group, without reaching statistical significance (**Figure 1**). Conversely, adiponectin levels were reduced in the serum fractions of patients [6.44 μg/mL (3.70; 10.12)] compared with control subjects [8.82 μg/mL (7.15;14.77), *p*= 0.013] (**Figure 1**).

### 3.3 Leptin and adiponectin oppositely correlate with psoriasis severity and body composition in female patients

To investigate the potential relationships among BIA parameters, serum adipokines and disease severity and the influence of sex on them, we conducted statistical analysis of association among PASI score, BIA measurements, lipid profile, and serum adipokine levels, by stratifying our study population into men and women.

**Figure 2:**
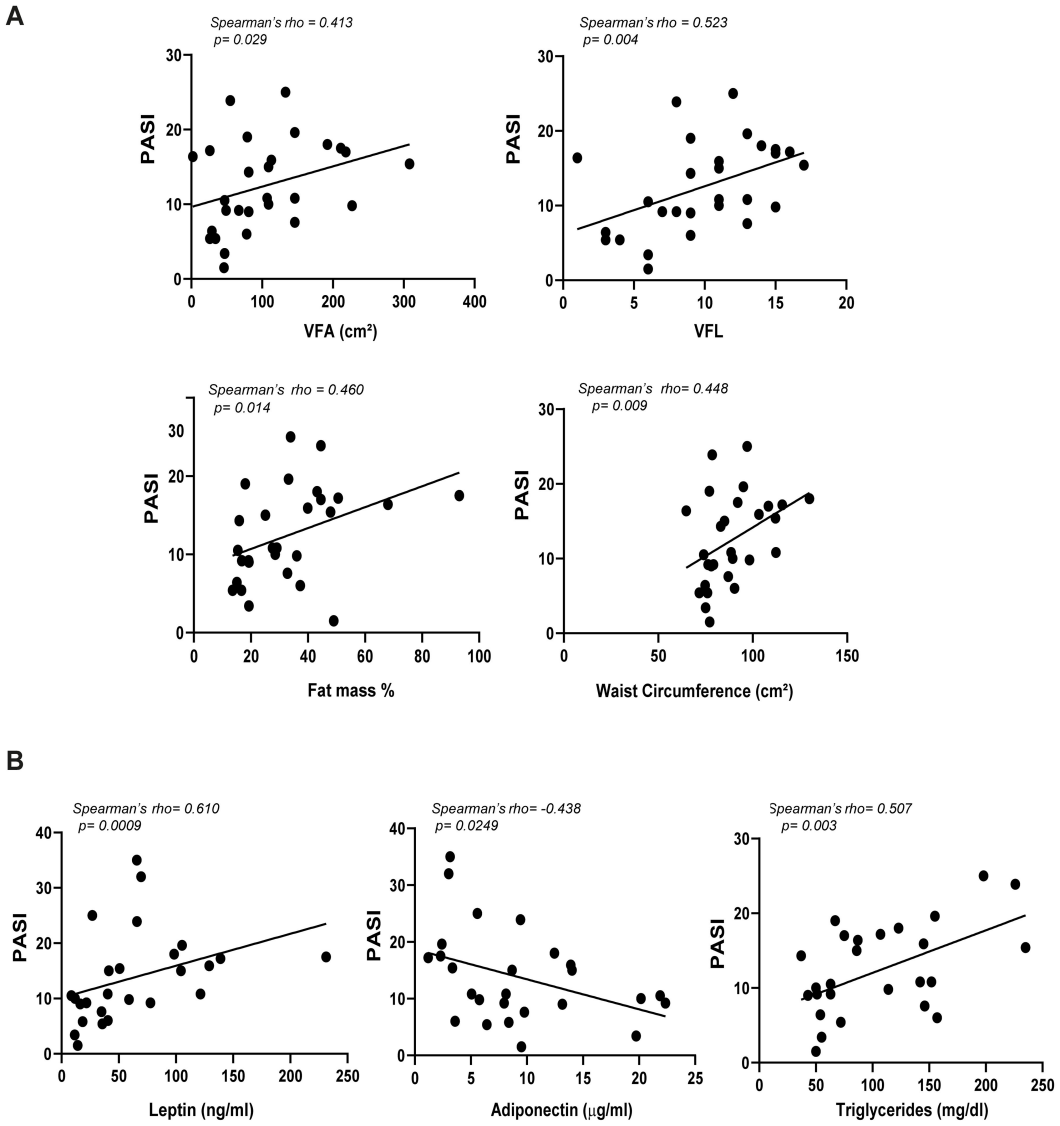
Correlations among PASI and adiposity indices **(A)**, and PASI and serum adipokines **(B)** in female patients with psoriasis (n=28). Spearman’ rho test was used for correlation analysis. r indicates the strength of the linear relationship, and p value shows the probability that the slope of the true relationship is zero. *p*≤ 0.05 was considered significant.

**Figure 3:**
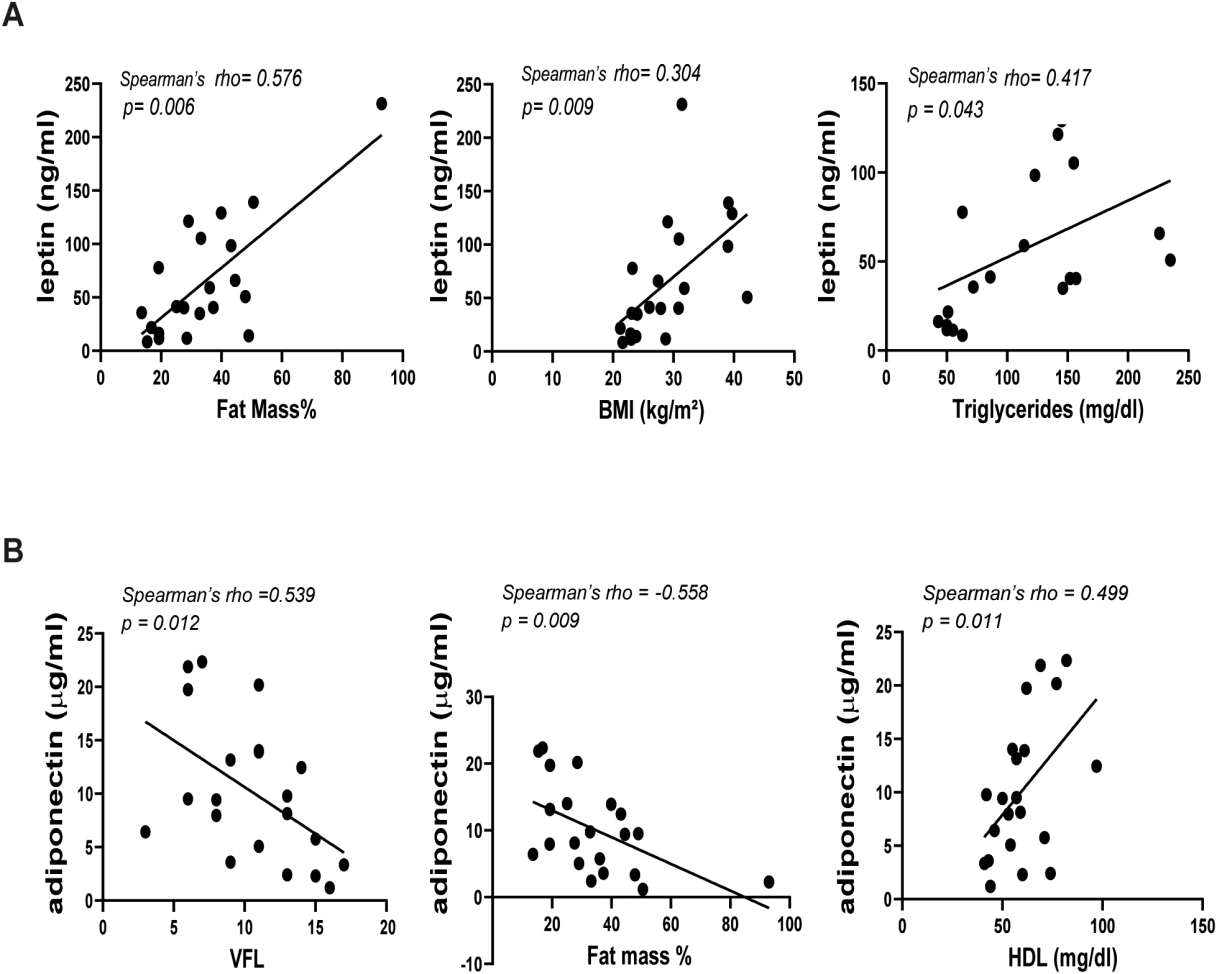
Correlations between leptin **(A)** or adiponectin **(B)** and BIA parameters in female patients with psoriasis (n=21). Spearman’ rho test was used for correlation analysis. *p* ≤ 0.05 was considered significant.

Comprehensive details on the correlation coefficients for men and women are represented in **Table 2** and **Table 3**, respectively. Notably, PASI correlated exclusively with fat mass in men (**Table 2**), while it showed correlations with several parameters in women. In detail, in women significant correlations were observed between disease severity expressed by PASI and body composition parameters, including VFA/VFL indices, fat mass and waist circumference (**Figure 2A**, **Table 3**). Notably, in female patients, a strong positive correlation was also observed between PASI score and serum leptin levels (rho= 0.610; *p*= 0.0009) (**Figure 2B**), according to its pro-inflammatory function. Consistently, baseline PASI also showed a significant positive correlation with VFL (rho= 0.523; *p*= 0.004) and triglycerides (rho= 0.507; *p*= 0.003) (**Figures 2A, B**). In addition, in this female subgroup, serum leptin levels exhibited positive correlations with both fat mass (rho= 0.576; *p*= 0.006) and triglycerides (rho= 0.417; *p*= 0.04, **Figure 3A**). Conversely, in line with its anti-inflammatory properties, adiponectin levels showed negative correlations with VFL (rho= -0.539; *p*= 0.012) and fat mass percentage (rho= -0.558; *p*= 0.009), while it exhibited a positive correlation with HDL cholesterol (rho= 0.499; *p*= 0.01), traditionally considered the “good cholesterol”, due to its association with low risk of atherosclerotic cardiovascular disease (27–29) (**Figure 3B**). However, the linear regression analysis confirmed a significant association between baseline PASI and fat mass (beta-standardized coefficient 0.115 [0.009-0.220], *p*=0.034), while no significant associations were found with sex, VFL, waist circumference, and adipokines. Multicollinearity among variables was low, with Variance Inflation Factors (VIF) values between 1 and 5 (**Supplementary Table S1**).

**Table 2:** Correlations among PASI, BMI, BIA parameters, lipidic profiles, and adipokine levels in men of the patient cohort.

	Baseline PASI	BMI (kg/m^2^)	VFA (cm^2^)	VFL	Fat Mass %	Total cholesterol (mg/dL)	HDL (mg/dL)	LDL (mg/dL)	Triglycerides (mg/dL)	Waist circumf. (cm)	VAI	Visfatin (ng/ml)	Leptin (ng/ml)	Adiponectin (µg/ml)
Baseline PASI	**1**	.086	.075	.206	,402**	.034	.04	.06	-.02	.086	-.017	.179	-.144	.252
		*.522*	*.622*	*.174*	*.006*	*.802*	*.766*	*.661*	*.882*	*.526*	*.903*	*.27*	*.377*	*.117*
BMI (kg/m^2^)		**1**	,643**	,718**	,425**	.018	-.131	-.115	,264*	,832**	,265*	.059	,613**	-.288
			*.000*	*.000*	*.004*	*.893*	*.332*	*.398*	*.047*	*.000*	*.048*	*.716*	*.000*	*.072*
VFA (cm^2^)			**1**	,751**	.321*	.077	-.026	-.083	,389**	,706**	,366*	.036	,455*	-.200
				*0*	*.031*	*.621*	*.869*	*.599*	*.009*	*.000*	*.015*	*.847*	*.01*	*.28*
VFL				**1**	.335*	.119	-.132	-.025	,490**	,858**	,499**	.076	,507**	-.014
					*.025*	*.443*	*.392*	*.873*	*.001*	*.000*	*.001*	*.684*	*.004*	*.939*
Fat Mass (%)					**1**	.111	-.086	.037	.258	,374*	.195	.086	.242	-.023
						*.473*	*.578*	*.811*	*.091*	*.011*	*.205*	*.645*	*.189*	*.901*
Total cholesterol (mg/dL) *p*						**1**	,344**	,885**	.146	-.003	-.037	.001	.175	.142
							*.009*	*.000*	*.277*	*.985*	*.788*	*.994*	*.281*	*.381*
HDL (mg/dL)							**1**	.071	-,291*	-.139	-,671**	-.199	-.061	.272
								*.601*	*.028*	*.307*	*.000*	*.218*	*.706*	*.090*
LDL (mg/dL)								**1**	-.021	-.136	-.044	-.024	.114	.101
									*.876*	*.322*	*.748*	*.886*	*.490*	*.539*
Triglycerides (mg/dL)									**1**	,302*	,868**	.178	.084	-.211
										*.024*	*.000*	*.271*	*.608*	*.191*
Waist circumf. (cm)										**1**	,370**	.042	,657**	-,318*
											*.005*	*.799*	*.000*	*.049*
VAI											**1**	.211	.151	-.314
												*.198*	*.357*	*.051*
Visfatin (ng/ml)												**1**	.021	.191
													*.897*	*.237*
Leptin (ng/ml)													**1**	-,318*
														*.046*
Adiponectin (µg/ml)														**1**

Results are reported as Spearman’s rho coefficients. Significant values (*p* < 0.05) are highlighted in gray.

**Table 3:** Correlations among PASI, BMI, BIA parameters, lipidic profiles, and adipokine levels in women of the patient cohort.

	Baseline PASI	BMI (kg/m^2^)	VFA (cm^2^)	VFL	Fat Mass %	Total cholesterol (mg/dL)	HDL (mg/dL)	LDL (mg/dL)	Triglycerides (mg/dL)	Waist circumf. (cm)	VAI	Visfatin (ng/ml)	Leptin (ng/ml)	Adiponectin (µg/ml)
Baseline PASI	**1**	.326	.413*	.523**	.460*	-.010	.083	-.196	.507**	.448**	.405*	-.130	.583**	-.462*
	.	*.064*	*.029*	*.004*	*.014*	*.955*	*.644*	*.282*	*.003*	*.009*	*.021*	*.537*	*.002*	*.020*
BMI (kg/m^2^)		**1**	.653**	.821**	.672**	.269	-.113	.144	.600**	.875**	.526**	-.194	.496*	-.442*
		.	*.000*	*.000*	*.000*	*.130*	*.532*	*.431*	*.000*	*.000*	*.002*	*.353*	*.012*	*.027*
VFA (cm^2^)			**1**	.824**	.341	.201	.229	-.052	.453*	.716**	.305	-.120	.373	-.235
				*.000*	*.075*	*.306*	*.241*	*.798*	*.018*	*.000*	*.122*	*.603*	*.096*	*.305*
VFL				**1**	.537**	.160	.054	.017	.518**	.896**	.429*	-.133	.613**	-.539*
					*.003*	*.417*	*.785*	*.933*	*.006*	*.000*	*.026*	*.564*	*.003*	*.012*
Fat Mass %					**1**	.082	-.005	-.040	.540**	.571**	.449*	-.016	.576**	-.558**
						*.677*	*.978*	*.841*	*.004*	*.002*	*.019*	*.947*	*.006*	*.009*
Total cholesterol (mg/dL) *p*						**1**	.316	.852**	.350*	.337	.158	.003	-.181	.083
							*.074*	*.000*	*.050*	*.055*	*.387*	*.988*	*.387*	*.692*
HDL (mg/dL)							**1**	.053	-.31	-.045	-.580**	.002	-.067	.499*
								*.773*	*.084*	*.805*	*.001*	*.993*	*.749*	*.011*
LDL (mg/dL)								**1**	.214	.207	.150	.040	-.211	-.027
									*.248*	*.255*	*.421*	*.848*	*.311*	*.900*
Triglycerides (mg/dL)									**1**	.581**	.937**	-.107	.417*	-.530**
										*.000*	*.000*	*.619*	*.043*	*.008*
Waist circumf. (cm)										**1**	.514**	-.180	.519**	-.459*
											*.003*	*.389*	*.008*	*.021*
VAI											**1**	-.103	.411*	-.612**
												*.630*	*.046*	*.001*
Visfatin (ng/ml)												**1**	-.199	.118
													*.340*	*.575*
Leptin (ng/ml)													**1**	-.623**
														*.001*
Adiponectin (µg/ml)														**1**

Results are reported as Spearman’s rho coefficients. Significant values (*p* < 0.05) are highlighted in gray.

### 3.4 Circulating leptin levels associate with comorbidities in female patients with psoriasis

Psoriasis is considered a systemic disease, frequently associated with several comorbidities (30). Indeed, it has been well-established that about 30% of patients with psoriasis have PsA (31). The prevalence of metabolic syndrome and hypertension is also higher in patients with psoriasis compared to healthy controls (32, 33).

In our study population, the most prevalent comorbidities were PsA (27.5%), diabetes (11%), and hypertension (27.5%). Notably, sex-specific analyses revealed distinct associations among BIA parameters, adipokines, and comorbidities. In detail, in men, none of the adipokines were associated with comorbidities (**Table 4A**). However, hypertension was positively associated with elevated values of VFA (213.6 ± 138.0 cm^2^, *p*=0.035), VFL (12.4 ± 4.3, *p*=0.047), percentage of fat mass (47.5 ± 24.3, *p*=0.019) and LDL (90.4 ± 44.2 ng/mL, *p*=0.049), compared to groups with lower values of the respective parameters (123.1 ± 73.8; 9.1 ± 3.5; 29.8 ± 21.4), respectively (**Table 4A**). In contrast, in women, mean levels of leptin were higher in patients with absence of PsA (80.7 ± 56.7 ng/mL, p=0.020) compared to those with PsA (36.7 ± 28.6 ng/mL), whereas they were positively associated with the presence of hypertension (108.9 ± 64.9 ng/mL vs 44.1 ± 29.1ng/mL (absence of hypertension), *p*=0.021) (**Table 4B**). A trend in positive association between leptin levels and diabetes was also observed, although it did nor result statistically significant. In female patients, the presence of hypertension was also associated with higher levels of BMI (32.2 ± 6.9kg/m^2^ vs 25.8 ± 5.4kg/m^2^ (absence of hypertension), *p*=0.021), and higher waist circumference values (102.5 ± 17.9cm vs 84.4 ± 11.9cm (absence of hypertension), *p*=0.011). Finally, high values of waist circumference were associated with the presence of diabetes (105.8 ± 20.1 cm vs 86.4 ± 13.3 cm (absence of diabetes), *p*=0.022).

**Table 4A:** Associations among PASI, BIA measurements, lipid profile, and adipokine levels according to the presence of comorbidities in men.

	Psoriatic Arthritis			Type 2 Diabetes			Hypertension		
	No	Yes	p*	No	Yes	p*	No	Yes	p*
	Mean (SD)	Mean (SD)		Mean (SD)	Mean (SD)		Mean (SD)	Mean (SD)	
Baseline PASI	17.4 (12.5)	12.6 (8.2)	*0.171*	15.8 (11.9)	19.9 (8.3)	*0.190*	16.4 (13.2)	15.3 (6.1)	*0.566*
BMI (kg/m^2^)	27.7 (5.9)	27.7 (4.4)	*0.540*	27.6 (5.3)	28.1 (8.6)	*0.750*	27.1 (4.9)	29.2 (6.9)	*0.347*
VFA (cm^2^)	150.5 (112.3)	128.9 (42.6)	*0.761*	139.4 (93.9)	227.3 (165.9)	*0.219*	123.1 (73.8)	213.6 (138.0)	*0.035*
VFL	9.8 (4.3)	10.3 (2.5)	*0.569*	9.7 (3.9)	13.0 (4.6)	*0.244*	9.1 (3.5)	12.4 (4.3)	*0.047*
Fat mass (%)	37.5 (25.4)	23.6 (8.8)	*0.240*	34.4 (23.5)	30.2 (20.5)	*0.785*	29.8 (21.4)	47.5 (24.3)	*0.019*
Total cholesterol (mg/dL)	177.6 (37.9)	187.3 (40.4)	*0.389*	182.3 (38.5)	157.0 (32.2)	*0.135*	184.4 (35.8)	168.0 (44.2)	*0.160*
HDL (mg/dL)	52.7 (18.3)	53.5 (14.3)	*0.717*	52.4 (17.5)	58.0 (13.4)	*0.247*	51.3 (12.9)	57.5 (25.8)	*0.772*
LDL (mg/dL)	105.9 (34.9)	11.4 (35.4)	*0.591*	110.0 (34.9)	80.3 (21.8)	*0.047*	113.6 (28.9)	90.4 (44.2)	*0.049*
Tryglicerides (mg/dL)	108.1 (43.5)	123.4 (77.2)	*0.800*	112.9 (55.2)	104.2 (44.4)	*0.724*	111.5 (58.3)	113.8 (41.2)	*0.441*
Waist circumference (cm)	97.5 (18.0)	101.3 (12.1)	*0.180*	97.6 (16.3)	107.8 (18.8)	*0.194*	96.0 (14.9)	104.9 (19.5)	*0.114*
VAI	3.1 (2.0)	3.6 (2.6)	*0.573*	3.3 (2.2)	2.9 (1.6)	*0.966*	3.3 (2.3)	3.2 (1.6)	*0.511*
Visfatin (ng/ml)	5.7 (4.3)	4.3 (3.7)	*0.520*	5.5 (4.3)	5.1 (4.2)	*0.964*	5.5 (4.4)	5.4 (3.7)	*0.950*
Leptin (ng/ml)	26.2 (30.0)	30.6 (30.0)	*0.791*	26.4 (27.9)	31.0 (47.8)	*0.417*	23.7 (25.8)	36.2 (39.0)	*0.349*
Adiponectin (μg/ml)	6.6 (3.5)	7.1 (4.2)	*0.850*	6.7 (3.7)	6.6 (1.5)	*0.588*	6.4 (3.5)	7.6 (3.8)	*0.281*

Data were reported as the mean value and standard deviation (SD) of the variables examined, in presence (Yes) or absence (No) of the specific comorbidity. Significant values are highlighted in gray. p* from Mann-Whitney U test.

**Table 4B:** Associations among PASI, BIA measurements, lipid profile, and adipokine levels according to the presence of comorbidities in women.

	Psoriatic Arthritis			Type II Diabetes			Hypertension		
	No	Yes	p*	No	Yes	p*	No	Yes	p*
	Mean (SD)	Mean (SD)		Mean (SD)	Mean (SD)		Mean (SD)	Mean (SD)	
Baseline PASI	14.3 (6.7)	13.6 (10.3)	*0.544*	13.5 (8.1)	17.4 (5.6)	*0.132*	13.1 (7.8)	16.8 (7.6)	*0.124*
BMI (kg/m^2^)	28.2 (6.7)	26.1 (5.9)	*0.422*	26.7 (6.1)	32.5 (6.7)	*0.063*	25.8 (5.4)	32.3 (6.9)	*0.021*
VFA (cm^2^)	110.1 (77.7)	88.6 (65.4)	*0.611*	100.7 (72.3)	124.0 (914)	*0.767*	96.5 (73.7)	122.6 (75.8)	*0.445*
VFL	10.3 (4.2)	8.6 (4.5)	*0.371*	9.2 (4.2)	13.2 (3.6)	*0.080*	8.9 (4.3)	12.0 (3.6)	*0.097*
Fat mass (%)	32.9 (18.6)	34.5 (17.9)	*0.647*	30.3 (13.9)	51.5 (30.7)	*0.101*	29.9 (14.3)	41.9 (24.1)	*0.178*
Total cholesterol (mg/dL)	182.7 (35.0)	183.6 (33.4)	*0.984*	184.0 (30.2)	177.4 (55.2)	*0.616*	184.5 (29.6)	179.0 (45.7)	*0.558*
HDL (mg/dL)	59.2 (13.0)	61.3 (16.7)	*0.891*	59.8 (12.8)	60.2 (21.5)	*0.563*	58.0 (11.3)	64.7 (19.5)	*0.585*
LDL (mg/dL)	101.8 (30.3)	99.5 (17.3)	*0.669*	104.3 (23.9)	83.6 (36.7)	*0.253*	104.0 (19.8)	92.2 (41.8)	*0.163*
Tryglicerides (mg/dL)	106.9 (57.8)	104.5 (63.7)	*0.760*	102.1 (56.0)	134.5 (77.9)	*0.319*	107.7 (65.5)	101.4 (33.3)	*0.728*
Waist circumference (cm)	89.2 (13.8)	89.7 (20.5)	*0.829*	86.4 (13.3)	105.8 (20.1)	*0.022*	84.4 (11.9)	102.5 (17.9)	*0.011*
VAI	3.6 (2.4)	3.8 (3.3)	*0.776*	3.5 (2.3)	5.4 (4.4)	*0.279*	3.8 (2.9)	3.4 (1.6)	*0.794*
Visfatin (ng/ml)	5.7 (2.7)	5.7 (2.9)	*0.692*	6.2 (2.6)	3.9 (2.3)	*0.103*	6.2 (3.1)	4.7 (0.8)	*0.221*
Leptin (ng/ml)	80.7 (56.7)	36.7 (28.6)	*0.020*	52.4 (37.6)	114.7 (76.7)	*0.057*	44.1 (29.1)	108.9 (64.9)	*0.021*
Adiponectin (μg/ml)	8.1 (6.4)	11.5 (6.2)	*0.126*	10.2 (6.6)	5.9 (4.5)	*0.118*	9.7 (6.2)	8.5 (7.1)	*0.440*

Data were reported as the mean value and standard deviation (SD) of the variables examined, in presence (Yes) or absence (No) of the specific comorbidity. Significant values are highlighted in gray. p* from Mann-Whitney U test.

### 3.5 Serum leptin levels strongly correlate with BMI in female patients

**Figure 4:**
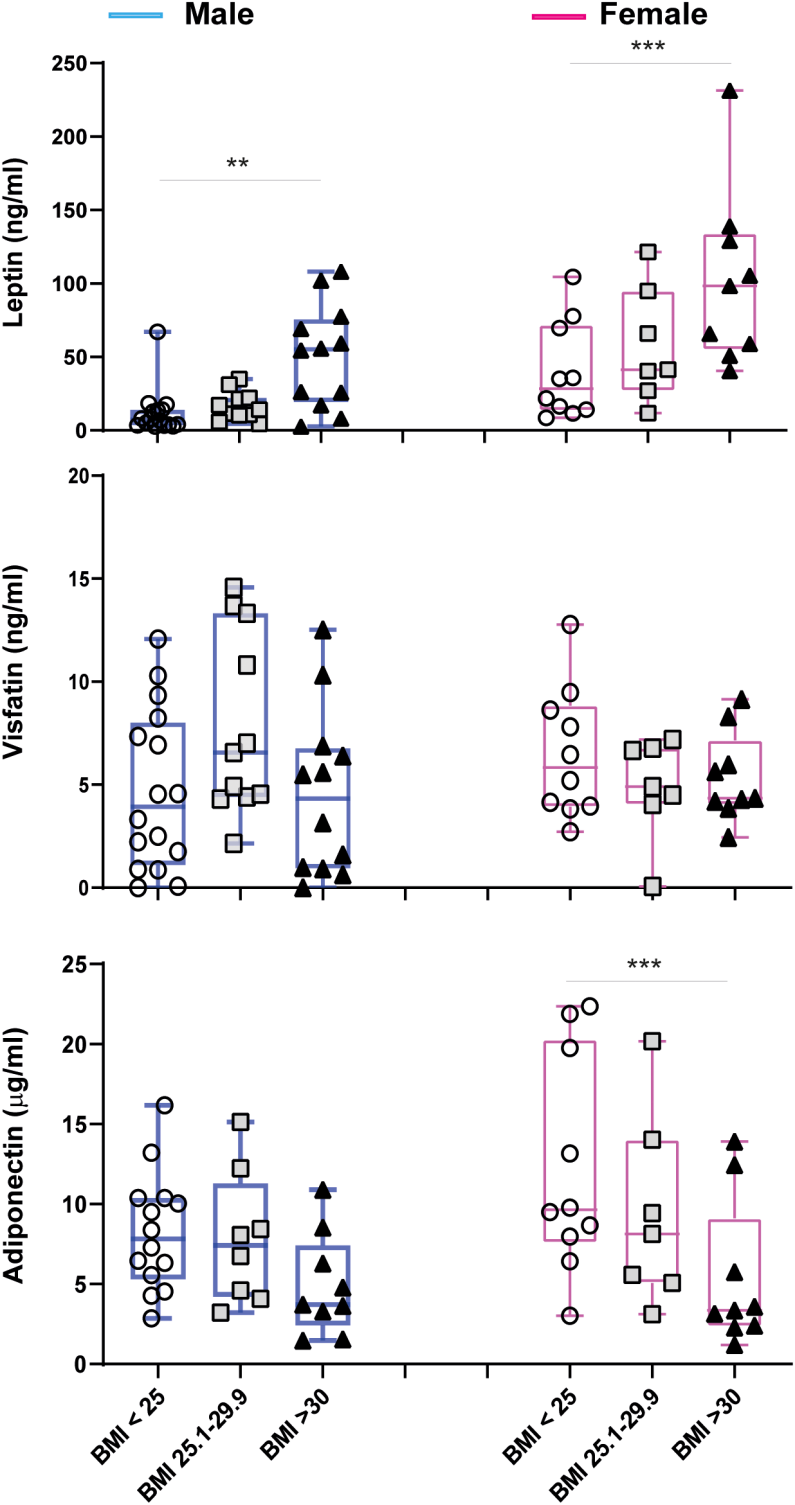
Circulating adipokine levels in male (blue) and female (pink) psoriatic patients, stratified for BMI values (male: BMI<25 n=16, BMI 25.1-29.9 n=11, BMI>30 n=12; female: BMI<25 n=10, BMI 25.1-29.9 n=7, BMI>30 n=9). Comparisons were made between BMI groups of the same gender with ANOVA 1 Way test or Kruskal Wallis Test, according to distribution of data. **p = 0.01; ***p = 0.001.

To further deepen the sex-related associations among BMI and adipokine levels, we stratified patients by sex and BMI into three groups: normal weight, overweight, and obese. This resulted in six distinct groups, as reported in **Figure 4**. In both men and women, leptin levels gradually increased with increasing BMI values, although this correlation was more significant in women (**Figure 4**). *Vice versa*, adiponectin levels tended to decrease with increasing BMI values and this association was more evident in women than in men, although it did not reach statistical significance in men for BMI>30. The analysis revealed no significant differences in visfatin levels between BMI groups in either women or men.

### 3.6 Anti-IL-23 biologic treatment reduces circulating visfatin levels in women with psoriasis

**Figure 5:**
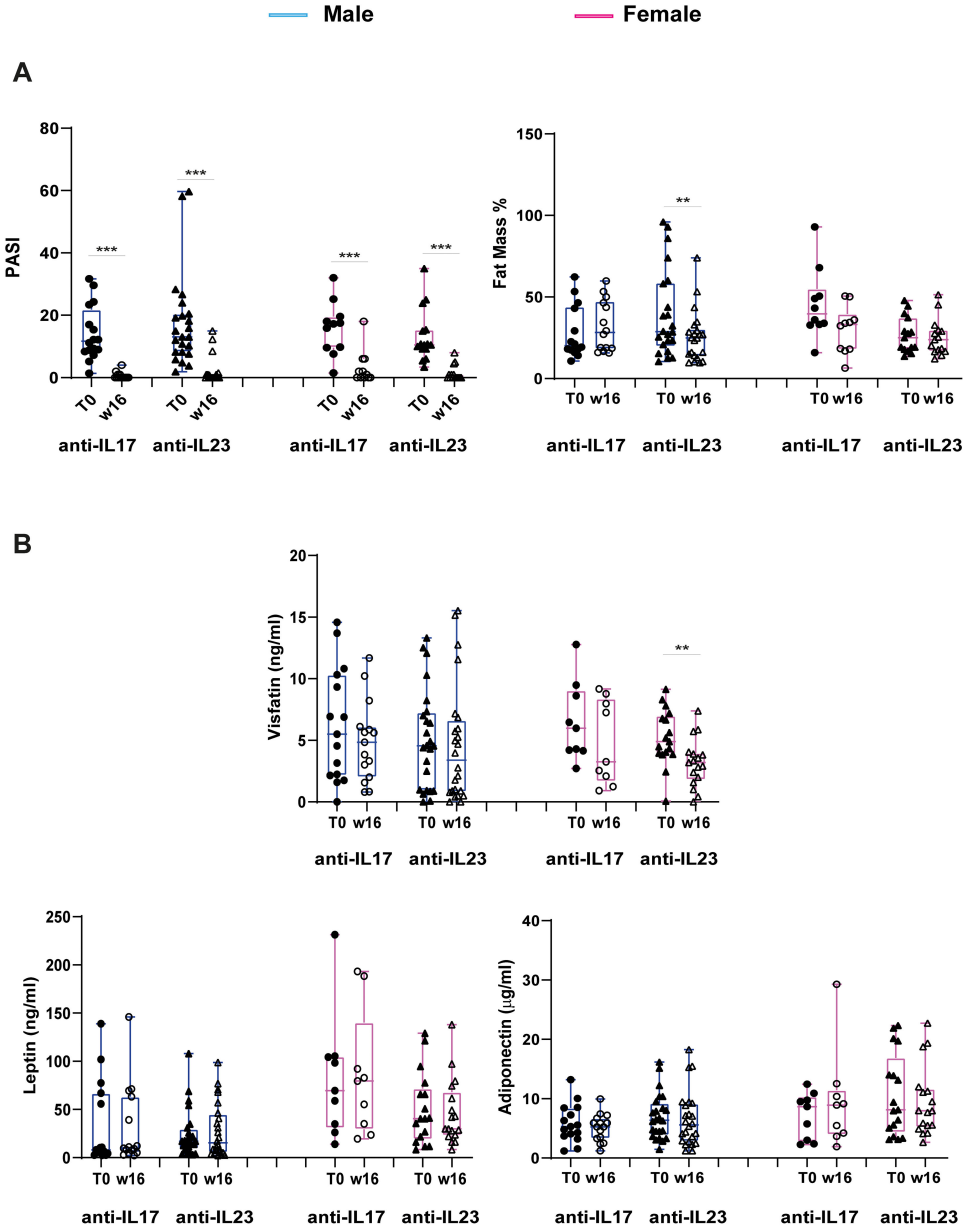
Changes in PASI scores, percentage of Fat Mass and adipokine levels at W16 of treatment with anti-IL17 or anti-IL-23 biologics in men and women. **(A)** Reduction of PASI scores (male: anti-IL-17 n=15, anti-IL-23 n=24; female: anti-IL-17 n=9, anti-IL-23 n=17) and Fat Mass % (male: anti-IL-17 n=14, anti-IL-23 n=22; female: anti-IL-17 n=10, anti-IL-23 n=15) at W16 of treatment with specific biologics. Statistical significance was assessed by Kruskal Wallis Test.***p <0.001, **p < 0.05. **(B)** Changes in visfatin, leptin and adiponectin levels in male and female patients undergone anti-IL-17 or anti-IL-23 biologic treatment (male: anti-IL-17 n=15, anti-IL-23 n=24; female: anti-IL-17 n=9, anti-IL-23 n=17). Statistical significance was assessed by Kruskal Wallis Test, Mann-Whitney *U* test and T-Test, respectively. **p < 0.05.

We next investigated the interaction of the distinct anti-IL-17 and anti-IL-23 biological treatments with body composition and circulating adipokine levels. To this end, patients were divided into two groups, based on the biological drugs they received and stratified for gender. **Figure 5** illustrates the changes in PASI scores and fat mass percentages, as well as in adipokine levels, before and after 16 weeks of biological treatment.

As expected, both anti-IL-17 and anti-IL-23 biologics led to a significant reduction in PASI score in both men and women (**Figure 5A**).

Notably, together with the improvement of disease severity, treatment with anti-IL-23 resulted in a significant decrease in fat mass percentage but only in men, whereas the effect was not observed in patients treated with anti-IL-17 (**Figure 5A**). With respect to the adipokine levels, a significant reduction was observed only in visfatin levels after 16 weeks of anti-IL-23 biologic treatment in only female patients (**Figure 5B**). No significant changes were observed for the other circulating adipokines (**Figure 5B**).

### 3.7 High levels of circulating leptin are associated with an inefficacious therapeutic response to anti-IL-23 biologics

We next analysed the relationships among BIA indices, lipid profiles and adipokine levels at baseline and clinical outcomes with biologics, evaluated in terms of PASI75, PASI90 and PASI100 achievements at W16 of biologics treatment. **Table 5** summarizes the BIA and adipokine levels data reported as mean (SD) and subdivided on the basis of achievement (yes) or not (no) of PASI75, PASI90, PASI100. We observed that achieving PASI75 at week 16 was linked to lower levels at baseline of triglycerides (*p*= 0.037) and/or waist circumference (*p* = 0.037), as well as VAI (*p* = 0.019)] (**Table 5**). Similarly, PASI 90 achievement at week 16 was significantly associated with lower levels of BMI (*p* = 0.024), VFA (*p* = 0.025), VFL (*p* = 0.013), total cholesterol (*p* = 0.047), triglycerides (*p* = 0.018), waist circumference (*p* = 0.003), VAI (*p* = 0.026), and leptin (*p* = 0.042) at baseline.

**Table 5:** BIA measurements, lipid profile, and circulating adipokines at baseline in relation to clinical response (PASI75, PASI90 and PASI100 achievement) after 16 weeks of treatment.

Variables at baseline	PASI75 (W16) with anti-IL-17+anti-IL-23			PASI90 (W16) with anti-IL-17+anti-IL-23			PASI100 (W16) with antiIL-17+anti-IL-23		
	*No (6)*	*Yes (57)*		*No (12)*	*Yes (51)*		*No (23)*	*Yes (40)*	
	Mean (SD)	Mean (SD)	p*	Mean (SD)	Mean (SD)	p*	Mean (SD)	Mean (SD)	p*
BMI (kg/m^2^)	31.0 (5.3)	27.4 (6.0)	*0.085*	31.0 (5.9)	27.0 (5.7)	*0.021*	29.2 (5.3)	26.9 (6.2)	*0.043*
VFA (cm^2^)	179.6 (104.9)	124.5 (9.31)	*0.161*	186.4 (115.9)	116.1 (84.9)	*0.023*	145.2 (103.9)	121.6 (90.0)	*0.314*
VFL	13.0 (2.5)	9.9 (3.9)	*0.065*	12.7 (2.9)	9.6 (3.9)	*0.013*	10.8 (3.6)	9.8 (4.0)	*0.344*
Fat mass (%)	35.0 (8.3)	33.9 (22.2)	*0.327*	39.7 (20.9)	39.0 (34.9)	*0.108*	45.6 (35.6)	36.1 (31.0)	*0.178*
Total cholesterol (mg/dL)	205.1 (39.0)	177.5 (33.7)	*0.100*	196.8 (33.8)	176.2 (34.3)	*0.048*	186.0 (30.2)	176.8 (37.4)	*0.267*
HDL (mg/dL)	57.3 (19.8)	53.6 (13.5)	*0.669*	56.5 (15.8)	53.3 (13.8)	*0.429*	56.3 (14.6)	52.5 (13.8)	*0.231*
LDL (mg/dL)	114.1 (39.7)	104.2 (28.7)	*0.643*	11.4 (34.5)	103.7 (28.7)	*0.329*	107.7 (27.2)	103.7 (31.5)	*0.657*
Triglycerides (mg/dL)	153.4 (57.3)	106.0 (49.4)	*0.037*	141.1 (50.7)	103.9 (49.9)	*0.018*	109.5 (50.7)	11.4 (52.9)	*0.858*
Waist circumference (cm)	107.6 (16.6)	93.9 (16.4)	*0.037*	106.9 (5.1)	92.4 (16.1)	*0.003*	100.5 (15.9)	92.2 (16.8)	*0.023*
VAI	5.5 (3.0)	3.3 (2.2)	*0.019*	4.7 (2.6)	3.3 (2.2)	*0.026*	3.5 (2.3)	3.6 (2.4)	*0.888*
Visfatin (ng/ml)	6.7 (4.4)	5.6 (3.6)	*0.482*	5.0 (3.6)	5.9 (3.7)	*0.294*	5.7 (3.8)	5.7 (3.6)	*0.819*
Leptin (ng/ml)	60.0 (41.3)	38.9 (43.6)	*0.087*	75.4 (68.2)	32.8 (31.2)	*0.042*	52.5 (56.2)	34.2 (33.3)	*0.222*
Adiponectin (µg/ml)	7.8 (4.4)	7.8 (5.1)	*0.815*	7.5 (5.5)	7.8 (5.0)	*0.624*	8.6 (5.6)	7.3 (4.7)	*0.445*

Patients were divided according to whether they achieved (Yes) or not (No) an improvement of PASI scores indicated in each column (PASI75, PASI90, PASI100).Values were reported as mean with standard deviation (SD). p* statistical significance was assessed from Mann-Whitney U Test. Statistically significant results are highlighted in gray.

However, these associations were less pronounced for PASI100 response, with significant differences observed only for BMI (*p* = 0.043) and waist circumference (*p* = 0.023) (**Table 5**). To investigate the potential influence of the body composition parameters at baseline on the clinical response to specific treatments, we divided patients into two groups according to the anti-IL-17 and anti-IL-23 biologic therapies received. Thus, we analyzed the associations among BMI, BIA parameters, lipid profile, or adipokine levels and clinical outcomes with biologics, evaluated in terms of PASI90 achievement at W16 of treatment (**Table 6**). Analysis revealed that failure to achieve a PASI90 response with anti-IL-23 was significantly associated with higher levels at baseline of BMI (*p* = 0.018), VFL (*p* = 0.030), waist circumference (*p* = 0.005), VAI (*p* = 0.041), and, of note, leptin levels (*p* = 0.016) (**Table 6**). The significant association of leptin with clinical outcome was confirmed by a logistic regression model which showed a significant negative association between achieving PASI90 at week 16 and baseline leptin levels, after adjusting for sex, BMI, and treatment type (**Supplementary Table 3**).

**Table 6:** BIA measurements, lipid profile, and circulating adipokines at baseline in relation to PASI90 achievement after 16 weeks of treatment.

Variables at baseline	PASI90 (W16) with anti-IL 17 biologic treatment			PASI90 (W16) with anti-IL 23 biologic treatment		
	*No (5)*	*Yes (21)*		*No (9)*	*Yes (37)*	
	Mean (SD)	Mean (SD)	p*	Mean (SD)	Mean (SD)	p*
BMI (kg/m^2^)	30.3 (6.8)	28.1 (7.4)	*0.495*	31.4 (5.8)	26.4 (4.6)	*0.018*
VFA (cm^2^)	154.8 (47.5)	125.8 (105.2)	*0.255*	212.7 (152.4)	110.8 (72.9)	*0.080*
VFL	12.2 (2.4)	10.2 (4.5)	*0.341*	13.2 (3.4)	9.2 (3.5)	*0.030*
Fat mass (%)	42.8 (29.5)	30.8 (16.3)	*0.290*	37.1 (12.8)	33.7 (24.0)	*0.174*
Total cholesterol (mg/dL)	188.2 (28.8)	172.6 (34.1)	*0.313*	201.6 (37.0)	178.3 (34.7)	*0.131*
HDL (mg/dL)	65.2 (18.6)	54.4 (12.7)	*0.151*	51.7 (12.6)	52.7 (14.5)	*0.978*
LDL (mg/dL)	93.4 (33.5)	98.3 (25.6)	*0.786*	121.4 (32.5)	106.5 (30.1)	*0.188*
Triglycerides (mg/dL)	142.7 (38.3)	107.9 (54.0)	*0.119*	140.4 (57.5)	101.6 (48.1)	*0.056*
Waist circumference (cm)	103.8 (15.2)	95.7 (19.6)	*0.162*	108.7 (15.7)	90.4 (13.6)	*0.005*
VAI	4.0 (1.5)	3.4 (2.2)	*0.374*	5.0 (2.9)	3.3 (2.3)	*0.041*
Visfatin (ng/ml)	3.4 (1.9)	6.8 (4.2)	*0.123*	5.8 (4.0)	5.3 (3.3)	*0.973*
Leptin (ng/ml)	85.2 (106.8)	42.1 (41.8)	*0.746*	70.5 (48.5)	27.2 (21.6)	*0.016*
Adiponectin (μg/ml)	7.0 (5.0)	6.1 (3.3)	*0.808*	7.8 (6.0)	8.8 (5.5)	*0.398*

Patients were divided according to whether they achieved (Yes) or not (No) an improvement of PASI scores indicated in each column (PASI75, PASI90, PASI100) separately for anti-IL-17 or anti-IL-23 biological drugs. Values are reported as mean with standard deviation (SD). p* Statistical significance was assessed from Mann-Whitney U Test. Statistically significant results are highlighted in gray.

Conversely, no significant association was found for anti-IL-17 treatments. Finally, after stratifying for sex, these associations did not result statistically significant, due to limited patient size related to subgroups.

## 4 Discussion

In this study, we investigated the intricate relationship between psoriasis, obesity, and adipokines, with a focus on the influence of these last on disease severity and the response to anti-IL-17 and anti-IL-23 biologic therapies (19, 23). Obesity is a complex and multifactorial condition with genetic, behavioural, and socioeconomic origins, associated with an increased risk of dyslipidemia, cardiovascular disease, and osteoarthritis (34–40).

In a recent epidemiological study, Wang et al. reported a global prevalence of 25% of obesity associated to psoriasis with a gender-specific prevalence rates of 23% for men and 38% for women (41). Specifically, we performed association analyses among anthropometric parameters, body composition indices, lipid profiles as well as serum levels of adipokines with pro-inflammatory (i.e. leptin and visfatin) or anti-inflammatory (i.e adiponectin) action (42, 43).

Our data establish that psoriatic patients exhibit distinct differences in body composition and adipokine profiles compared to healthy controls. Specifically, patients with psoriasis presented with higher BMI, waist circumference, fat mass, and visfatin levels, alongside lower adiponectin levels. Although leptin levels tended to be higher in psoriatic patients, they did not reach the statistical significance. These findings corroborate previous research indicating a higher prevalence of overweight and obesity in individuals with psoriasis. The elevation of pro-inflammatory adipokines like visfatin, coupled with the reduction in anti-inflammatory adiponectin, lends further support to the concept of a chronic low-grade inflammatory state characteristic of psoriasis, potentially serving as a crucial link between obesity and disease pathogenesis (17, 44, 45).

**Figure 6:**
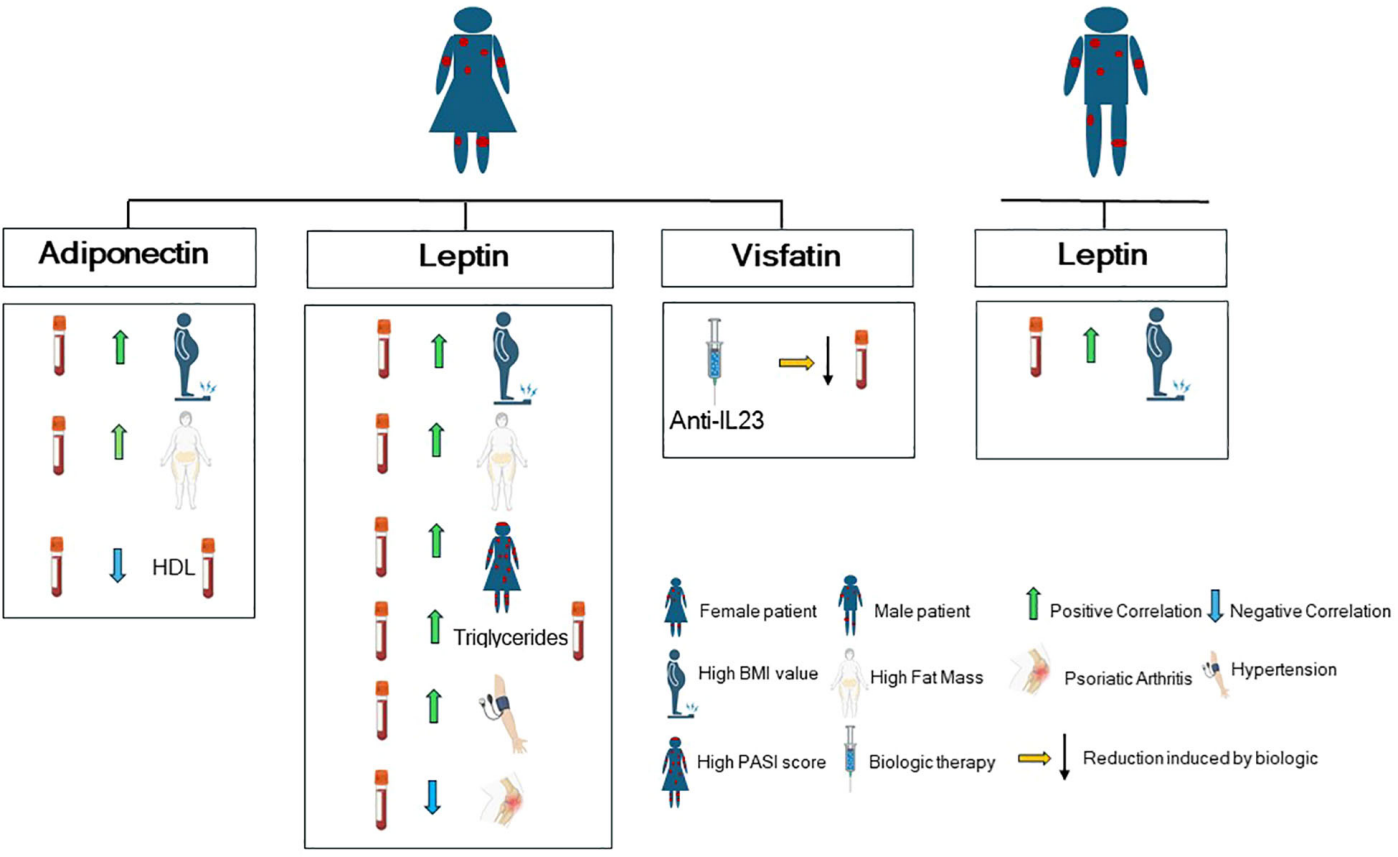
Graphical cartoon summarizing the correlation patterns between adipokines, comorbidities and metabolic parameters by sex stratification in our study population (with Biorender.com).

In line with previous studies (37, 38, 46, 47), we further observed that psoriasis severity significantly correlates with body composition parameters, in particular with visceral fat and fat mass (encompassing both visceral and subcutaneous fat) in our study population. Of note, none of the adipokines examined are associated with PASI in the overall study population, in line with existing data showing a very weak negative correlation of the only adiponectin with disease severity in a large pooled plaque psoriasis study population (48). Of note, our study uncovers important sex-specific differences in the correlation among adipokines, body composition indices, psoriasis severity and comorbidities. **Figure 6** summarizes the correlation patterns between adipokines, body composition indices, comorbidities and metabolic parameters by sex stratification.

Specifically, by stratifying our patient population for sex, we found that men exhibit a higher values of waist circumference and visceral fat than women. Accordingly, significant sex-specific differences exist in adipose tissue distribution and systemic inflammation, with women typically exhibiting more subcutaneous adipose tissue (SAT) and less visceral adipose tissue (VAT) than men (49), a phenomenon known as “sexual dimorphism of obesity” (50). In addition, we observed in women a positive correlation between PASI score and visceral fat indices, fat mass, waist circumference, and leptin levels, suggesting a stronger association between adiposity and disease severity in females. Conversely, adiponectin exhibited an inverse correlation with fat mass and visceral fat, suggesting a close association between these two adipokines and adipose tissue, which may predispose individuals, particularly women, to an increased risk of psoriasis onset.

Adipokine expression is influenced by several factors. In particular, serum leptin levels positively correlate with fat mass, but they are influenced also by sex steroids and fat distribution (51–54).

There is a differential expression of leptin in subcutaneous and visceral adipose tissue (55). *In vitro* studies have demonstrated that leptin secretion from SAT is significantly higher than from intra-abdominal fat, in both obese and non-obese individuals (56). Because subcutaneous fat comprises 80% of total body fat, SAT is considered to be the most important source of leptin (55).

In our cohort of psoriatic patients, serum leptin levels increased with increasing BMI more significantly in women than in men, suggesting an important influence of gender on its release. Thus, the prevalence of SAT in women could explained, at least in part, the higher levels of leptin in women with psoriasis compared to affected men observed in our study population. However, leptin production and release can be strongly influenced by estrogens that can stimulate leptin secretion in adipose tissue (57). Of note, adipose tissue itself releases estrogens. Indeed, adipose tissue contains the enzyme aromatase, which converts androgens (like testosterone) into estrogens. This is a significant source of estrogen, particularly in postmenopausal women, whose ovaries produce less estrogen (58). However, both subcutaneous and visceral adipose tissue can produce aromatase and thus estrogens (59).

Adipose tissue is now recognized also as a component of the innate immune system and adipokines play a significant role in the pathogenesis of insulin resistance and metabolic syndrome. They are also associated with various complications, including dyslipidemia, diabetes mellitus, hypertension, and premature cardiovascular disease (5). These comorbidities are more prevalent in psoriatic patients compared to the general population (23, 60).

Our study shows that leptin levels are associated with hypertension and, at lower extent, with diabetes, particularly in women, highlighting a potential link between leptin, metabolic comorbidities, and psoriasis, with a more pronounced effect in women. These sex-specific findings underscore the importance of considering gender as a critical factor in the assessment and management of psoriasis and its associated comorbidities.

Furthermore, our study explored the effects of anti-IL-17 and anti-IL-23 biologic treatments on adipokine levels and body composition. Currently, several biologic agents are approved for the treatment of moderate to severe psoriasis (4). IL-17 inhibitors are a class of biologics that target the IL-17 ligand (or its receptor), an essential pro-inflammatory cytokine, which is mainly secreted by Th17 cells and subsets of innate lymphoid cells (61).

Anti-IL-23 inhibitors act by blocking the p19 subunit of IL-23, preserving the IL-12/Th1 axis, which is relevant for host immunity, while interfering with the differentiation of Th17 cells (62). Both anti-IL-17 and anti-IL-23 biologics effectively reduced PASI scores after 16 weeks, confirming their efficacy in treating psoriasis (63). Notably, anti-IL-23 treatment, but not anti-IL-17, led to a significant reduction in fat mass only in men, indicating a potential differential effect of these biologics on metabolic parameters. The reduction in fat mass was not accompanied by a reduction in body weight or visceral fat, leading to speculation that it might be related to a reduction in subcutaneous fat in these patients. However, to date, there is no evidence to support this hypothesis.

Additionally, visfatin levels decreased significantly following both treatments, with a more pronounced reduction observed in women treated with anti-IL-23, suggesting a sex-specific modulation of visfatin by anti-IL-23 therapy. The decrease in circulating visfatin levels after biological treatment reinforces its significant role in the pathogenesis of psoriasis, as previously described (17), thus identifying it as an obesity-independent biomarker of psoriasis. Indeed, visfatin, also known as nicotinamide phosphoribosyltransferase (NAMPT) is an enzyme with a dual entity: an intracellular form (iNAMPT), implicated in NAD biosynthesis, and an extracellular form (eNAMPT), with pro-inflammatory properties in several metabolic and inflammatory diseases (64–66). Our findings also provide further insights into the potential metabolic effects of biologic therapies and highlight the need for investigation into their long-term impact on adipokine profiles and body composition.

In our study we found that the achievement of PASI75, PASI90, and PASI100 at week 16 of biological treatments was associated with lower levels of body composition parameters (BMI, waist circumference, VFA, VFL) and lipid profile (triglycerides and total cholesterol). This observation aligns with existing literature data indicating that excess adipose tissue can negatively impact the therapeutic response to both traditional and biologic agents in psoriasis patients (24, 60, 67).

One of the most significant findings of this study is the association between higher baseline leptin levels with a poorer PASI90 response to anti-IL-23 biologic treatment. This suggests that leptin could potentially serve as a predictive biomarker for treatment response, particularly for anti-IL-23 biologics. Patients with elevated levels of leptin may benefit from more intensive management of metabolic risk factors or consideration of alternative treatment strategies.

This study has some limitations. The observational nature of the study design limits the ability to establish causality between adipokines and psoriasis, or treatment response. Additionally, the follow-up period of 16 weeks might not be sufficient to fully capture the long-term effects of biologic treatments on adipokine levels and metabolic parameters, and to confirm leptin as a predictive factor of response to anti-IL-23 biologic. Further long-term observations will be carried out in order to validate leptin as a durable predictive biomarker. Longitudinal studies with larger sample sizes and diverse ethnic backgrounds are needed to confirm these findings and investigate the long-term impact of biologic therapies on metabolic health. Further research is needed to elucidate the mechanisms underlying the observed associations between adipokines, psoriasis severity, and treatment response. In particular, future studies should be addressed to investigate the expression of adipokines locally in the psoriatic skin, and to associate it with the body composition parameters and the histopathological hallmarks of psoriatic lesions. Notably, exploring the potential therapeutic implications of targeting adipokines in psoriasis management may also be valuable.

In conclusion, a comprehensive assessment of body composition, adipokine levels, and metabolic parameters should be considered in the management of psoriasis, particularly in women. The study highlights the potential of leptin as predictive marker for treatment response to anti-IL-23 biologics. This finding could aid in personalizing treatment decisions and optimizing therapeutic strategies for patients with psoriasis. Finally, the observed differential effects of anti-IL-17 and anti-IL-23 biologics on metabolic parameters warrant further investigation to fully elucidate their long-term metabolic consequences and inform clinical practice.

## Data Availability

The datasets presented in this article are not readily available because the clinical databases are deposited in IDI-IRCCS. Requests to access the datasets should be directed to Stefania Madonna, s.madonna@idi.it.
