# Supplementary figures and images for: Leptin: a gender and obesity-related marker predictive of metabolic comorbidities and therapeutic response to anti-IL-23 biologic drugs in psoriatic patients
Source: Front Immunol. 2025 Jul 16;16:1607312. doi: 10.3389/fimmu.2025.1607312 (PMC12307160; doi:10.3389/fimmu.2025.1607312)

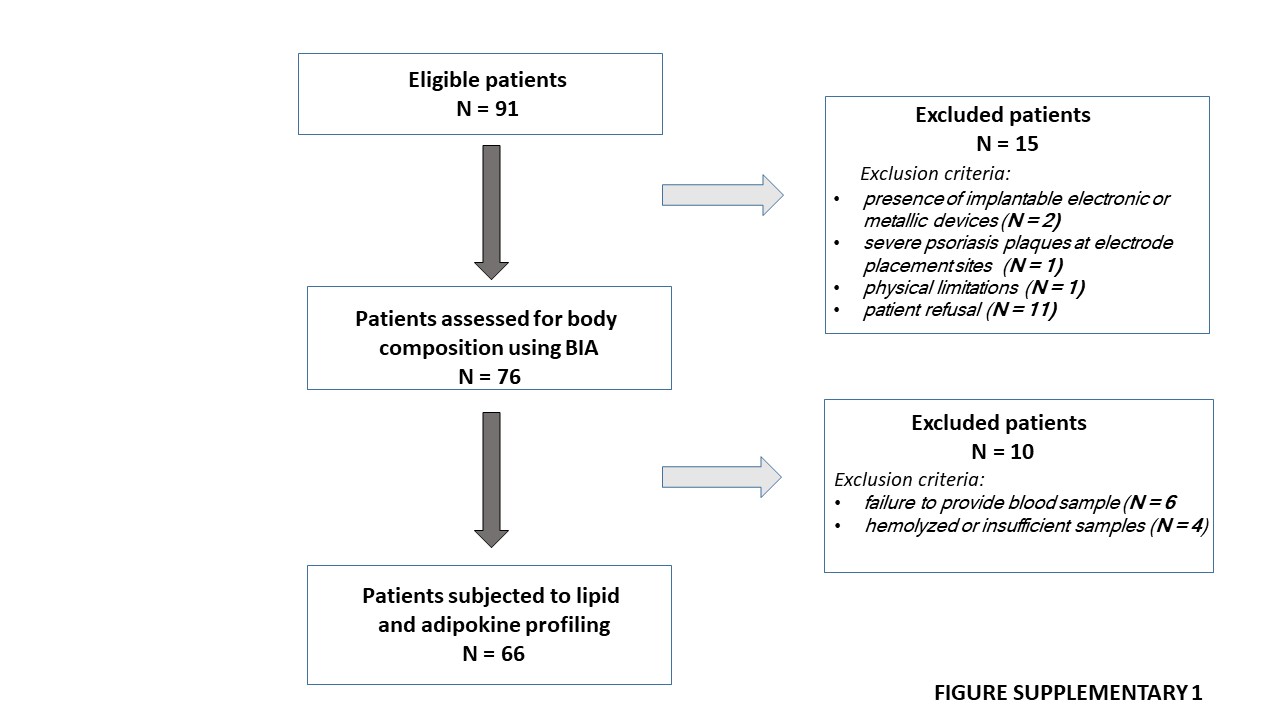

Supplement: Supplementary file 1 [file Image1.jpg]
