# Supplementary material for: Leptin: a gender and obesity-related marker predictive of metabolic comorbidities and therapeutic response to anti-IL-23 biologic drugs in psoriatic patients
Source: Front Immunol. 2025 Jul 16;16:1607312. doi: 10.3389/fimmu.2025.1607312 (PMC12307160; doi:10.3389/fimmu.2025.1607312)
Supplement: Supplementary file 2 [file Table1.docx]

|  | Beta standardized coefficient | 95%CI  Lower limit | 95%CI  Upper limit | p |
| --- | --- | --- | --- | --- |
| Sex (M=1, F=2) | -4.785 | -10.371 | .802 | .091 |
| Visceral Fat Level (VFL) | .595 | -.187 | 1.378 | .132 |
| Fat Mass (%) | .115 | .009 | .220 | **.034** |
| Waist circumference (cm) | -.053 | -.236 | .131 | .568 |
| Visfatin (ng/ml) | .491 | -.090 | 1.071 | .096 |
| Leptin (ng/ml) | .026 | -.041 | .092 | .445 |
| Adiponectin (μg/ml) | .286 | -.165 | .737 | .208 |

**Table S1**

Linear regression model with PASI at baseline as the dependent variable
