# Supplementary material for: Leptin: a gender and obesity-related marker predictive of metabolic comorbidities and therapeutic response to anti-IL-23 biologic drugs in psoriatic patients
Source: Front Immunol. 2025 Jul 16;16:1607312. doi: 10.3389/fimmu.2025.1607312 (PMC12307160; doi:10.3389/fimmu.2025.1607312)
Supplement: Supplementary file 3 [file Table2.docx]

|  | **TOTAL (n=70)** | | | **MEN (n=43 )** | | | **WOMEN (n= 27)** | | |
| --- | --- | --- | --- | --- | --- | --- | --- | --- | --- |
| **Variable** | **T0** | **w16** | ***p **** | **T0** | **w16** | ***p**** | **T0** | **w16** | ***p**** |
| Baseline PASI | 15.9 (11.1) | 1.4 (3.1) | ***<0.001*** | 16.8 (12.6) | 1.3 (3.2) | ***<0.001*** | 14.4 (8.3) | 1.6 (3.1) | ***<0.001*** |
| BMI (kg/m^2^) | 27.8 (6.1) | 27.9 (6.1) | *0.703* | 27.8 (5.9) | 27.9 (5.7) | *0.724* | 27.8 (6.4) | 28.0 (6.8) | *0.936* |
| VFA (cm) | 129.3 (95.9) | 136.6 (100.7) | *0.858* | 146.1 (107.4) | 145.2 (98.9) | *0.614* | 105.2 (71.8) | 124.2 (104.0) | *0.846* |
| VFL | 10.1 (4.0) | 10.0 (4.4) | *0.754* | 10.1 (4.1) | 10.1 (4.1) | *0.924* | 10.1 (4.0) | 10.0 (4.8) | *0.499* |
| Fat mass % | 34.7 (21.5) | 27.7 (14.4) | ***0.018*** | 35.0 (23.7) | 28.2 (15.8) | *0.119* | 34.3 (18.3) | 27.1 (12.5) | *0.074* |
| Total cholesterol  (mg/dL) | 180.0 (35.6) | 178 (41.8) | *0.868* | 178.8 (36.7) | 177.4 (44.8) | *0.900* | 183.7 (35.5) | 179.6 (37.9) | *0.658* |
| HDL  (mg/dL) | 60.9 (55.4) | 54.9 (14.4) | *0.507* | 50.8 (14.4) | 53.3 (13.7) | *0.204* | 59.0 (13.7) | 57.1 (15.3) | *0.580* |
| LDL  (mg/dL) | 106.8 (29.1) | 102.1 (31.7) | *0.324* | 109.9 (29.5) | 104.8 (33.4) | *0.629* | 102.4 (28.4) | 98.3 (29.5) | *0.204* |
| Triglycerides  (mg/dL) | 107.2 (50.3) | 107.4 (48.9) | *0.958* | 109.4 (46.3) | 113.9 (48.8) | *0.757* | 103.5 (57.0) | 97.2 (48.3) | *0.559* |
| Waist circumference  (cm) | 95.2 (17.3) | 94.0 (17.3) | *0.345* | 98.5 (17.5) | 97.9 (18.5) | *0.700* | 90.1 (15.8) | 88.0 (13.6) | *0.209* |
| VAI | 3.5 (2.4) | 3.2 (1.9) | *0.479* | 3.3 (2.1) | 3.0 (1.7) | *0.780* | 3.7 (2.8) | 3.4 (2.2) | *0.587* |
| Visfatin (ng/ml) | 5.6 (3.7) | 4.3 (3.7) | ***0.009*** | 5.5 (4.2) | 4.6 (4.2) | *0.214* | 5.7 (2.7) | 3.9 (2.6) | ***0.006*** |
| Leptin (ng/ml) | 41.5 (43.7) | 41.4 (42.9) | *0.792* | 28.9 (29.6) | 27.5 (27.8) | *0.809* | 64.8 (52.4) | 63.6 (53.1) | *0.376* |
| Adiponectin (µg/ml) | 7.7 (5.0) | 7.4 (5.4) | *0.242* | 6.7 (3.6) | 6.1 (3.8) | *0.188* | 9.3 (6.4) | 9.4 (6.8) | *0.861* |
