# Supplementary material for: Leptin: a gender and obesity-related marker predictive of metabolic comorbidities and therapeutic response to anti-IL-23 biologic drugs in psoriatic patients
Source: Front Immunol. 2025 Jul 16;16:1607312. doi: 10.3389/fimmu.2025.1607312 (PMC12307160; doi:10.3389/fimmu.2025.1607312)
Supplement: Supplementary file 4 [file Table3.docx]

**Table S3**

Logistic regression model with PASI90 as the dependent variable.

|  | Exp (B) | 95%CI  Lower limit | 95%CI  Upper limit | p |
| --- | --- | --- | --- | --- |
|  |  |  |  |  |
| Sex (M vs F) | 0.833 | 0.180 | 3.854 | 0.815 |
| BMI | 0.965 | 0.849 | 1.096 | 0.579 |
| Leptin (ng/ml) | 0.980 | 0.961 | 0.999 | 0.044 |
| Treatment (IL17 vs IL23) | 2.093 | 0.432 | 10.156 | 0.359 |
